# Molecular docking of potential inhibitors with the mTOR protein

**DOI:** 10.6026/97320630017212

**Published:** 2021-01-31

**Authors:** JH Shazia Fathima, Jayaraman Selvaraj, Venkatacalam Sivabalan, Umapathy Vidhya Rekha, Rajagopal Ponnulakshmi, Veeraraghavan Vishnupriya, Malathi Kullappan, Radhika Nalinakumari Sreekandan, Surapaneni Krishna Mohan, Periyasamy Vijayalakshmi

**Affiliations:** 1 Department of Oral and Maxillofacial Pathology, Ragas Dental College and Hospitals, Chennai, India; 2 Department of Biochemistry,Saveetha Dental College and Hospitals, Saveetha Institute of Medical and Technical Sciences, Saveetha University, Chennai - 600 077, India; 3 Department of Biochemistry, KSR Institute of Dental Sciences and Research, Thiruchengodu-637215, Indi; 4 Department of Public Health Dentistry, Sree Balaji Dental College and Hospital, Pallikaranai, Chennai-600 100, India; 5 Central Research Laboratory, Meenakshi Academy of Higher Education and Research (Deemed to be University), Chennai-600 078, India; 6 Department of Research, Panimalar Medical College Hospital & Research Institute, Varadharajapuram, Poonamallee, Chennai - 600 123, India; 7 Department of Clinical Skills & Simulation, Panimalar Medical College Hospital & Research Institute, Varadharajapuram, Poonamallee, Chennai - 600 123, India; 8 Department of Biochemistry and Department of Clinical Skills & Simulation, Department of Research, Panimalar Medical College Hospital & Research Institute, Varadharajapuram, Poonamallee, Chennai - 600 123; 9 DBT-BIF Centre, PG & Research Department of Biotechnology & Bioinformatics, Holy Cross College (Autonomous), Trichy, Tamilnadu, India

**Keywords:** mTOR, paclitaxel, analogues, molecular docking, oral cancer

## Abstract

The mTOR (mammalian or mechanistic Target of Rapamycin) is linked with oral cancer. Therefore, it is of interest to study the molecular docking-based binding of paclitaxel (a FDA approved drug for oral cancer) and its analogues with mTOR. Hence, we report the binding features of 10-Deacetyltaxol, 7-Epi-10-deacetyltaxol, 7-Epi-Taxol and 6alpha-Hydroxypaclitaxel with mTOR for further consideration.

## Background:

The mTOR (mammalian or mechanistic Target of Rapamycin) is linked with oral cancer [1-8]. Therefore, it is of interest to study the molecular docking-based binding of paclitaxel (a FDA approved drug for oral cancer) and its analogues with mTOR.

## Materials and Methods:

### Ligand preparation:

Structure of paclitaxel and its 10 analogues were downloaded from PUBCHEM database in SDF format (Table 1 - see PDF).

### Protein Preparation:

A crystal structure of mTOR (PDB ID: 4JSV) was downloaded from the Protein Data Bank (PDB).

### Molecular Docking:

Molecular docking analysis has been performed using the Autodock module available in PyRx Version 0.8 [9-10] and visualized by PyMOL [11].

## Results and Discussion:

Paclitaxel and its 10 analogues were docked to the active site of mTOR and the desirable conformations of the studied ligands were identified. It is observed that the ligands were appropriately bound to the active site and in some instances has identical orientations and is equivalent to the typical drug used as control. The values of the binding energies are given in Table 2 (see PDF). These analogues were arranged in order based on binding energies; 10-Deacetyltaxol>7-Epi-10-deacetyltaxol>7-Epi-Taxol> 6alpha-Hydroxypaclitaxel. GLY-2203, THR-2207, ASP-2212,T HR-2214, SER-2221, ARG-2224, ARG-2234, LYS-2352 were the residues for hydrogen bond interactions with the ligands. This is similar to the interaction with paclitaxel. The binding energy of -6.8kcal/mol was shown by the docked findings of Paclitaxel with mTOR protein and the three hydrogen bond interaction was formed with GLY-2203, ARG-2224 & THR-2207 amino acid residues. Of the ten analogues, 10-Deacetyltaxol, 7-Epi-10-deacetyltaxol, 7-Epi-Taxol, and 6alpha-Hydroxypaclitaxel showed comparable effects to Paclitaxel as compared with other analogues.

**Figure 1:**
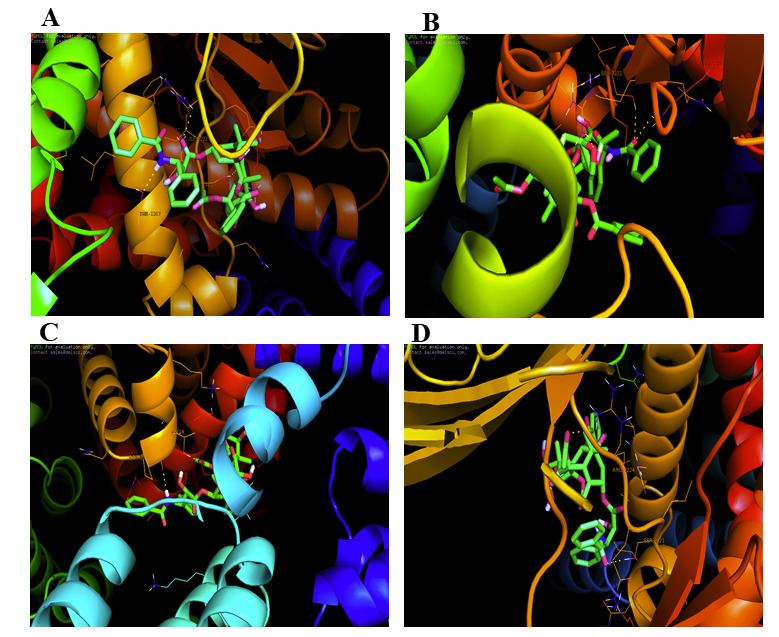
Molecular docking interaction of mTOR with (a) 10-deacetyltaxol; (b) 7-epi-10-deacetyltaxol; (c) 7-epi-taxol; (d) 6-alpha-hydroxypaclitaxel

10-Deacetyltaxol was chosen as the best ligand docked on the active mTOR segment with a binding energy of -6.7 kcal/mol (Table 2 - see PDF). This docking showed that 10-Deacetyltaxol was observed to be binding on the protein in the active segment due to the formation of three hydrogen bonds with THR-2207, SER-2221, ARG-2224 at a distance of 2.3Å, 2.5Å, 2.6Å and 2.3Å respectively (Figure 1). The best compound 10-Deacetyltaxol selected also showed the very same interaction of the hydrogen bond with THR-2207, almost equivalent to Paclitaxel with ARG-2224. The results obtained from molecular docking indicate that selected active compound 10-Deacetyltaxol can inhibit the growth of the cancer cell lines by inhibiting the mTOR. Orientation, and interaction of the ligand with the mTOR protein, paclitaxel, the standard FDA-approved drug used for the treatment of oral cancer, was docked. There were some good similarities when comparing the position, orientation, and interaction of the ligand (paclitaxel) with the topmost docked ligand conformation (10-Deacetyltaxol). This study showed that all paclitaxel analogues are more effective in targeting mTOR, particularly 10-Deacetyltaxol with binding energy, than the standard drug paclitaxel.

## Conclusion

We report the binding features of 10-Deacetyltaxol, 7-Epi-10-deacetyltaxol, 7-Epi-Taxol and 6alpha-Hydroxypaclitaxel with mTOR for further consideration.
